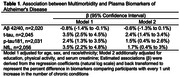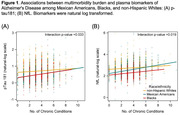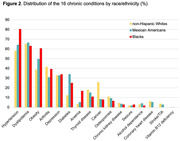# Association of Multimorbidity and Plasma Biomarkers of Alzheimer’s Disease: The HABS‐HD Study

**DOI:** 10.1002/alz.090553

**Published:** 2025-01-09

**Authors:** Xiaqing Jiang, Sid E. O'Bryant, Robert A Rissman, Leigh A. Johnson, Carrie B. Peltz, Kristine Yaffe

**Affiliations:** ^1^ University of California, San Francisco, San Francisco, CA USA; ^2^ University of North Texas Health Science Center, Fort Worth, TX USA; ^3^ University of California, San Diego, La Jolla, CA USA; ^4^ NCIRE‐The Veterans Health Research Institute, San Francisco, CA USA; ^5^ San Francisco Veterans Affairs Health Care System, San Francisco, CA USA; ^6^ Departments of Psychiatry and Behavioral Sciences, Neurology, and Epidemiology, University of California San Francisco, San Francisco, CA USA

## Abstract

**Background:**

Multimorbidity, known as the coexistence of two or more chronic conditions in the same individual, is prevalent among older adults and has been linked to an increased risk of dementia. Yet, little is known about its relationship with plasma Alzheimer’s disease (AD) biomarkers, especially in diverse populations. In the Health and Aging Brain Study: Health Disparities (HABS‐HD), we investigated the association of multimorbidity burden and plasma AD biomarkers.

**Method:**

We cross‐sectionally studied a diverse cohort of 2,110 dementia‐free participants (63% women, 43% Mexican Americans [MA], 44% non‐Hispanic Whites [NHW], and 13% Blacks) aged ≥50 years (mean age 65.6±8.5) at baseline (2017‐2023). Plasma AD biomarkers, including β‐amyloid (Aβ)42/40, total and phosphorylated tau (t‐tau and p‐tau181), and neurofilament light (NfL), were assayed using ultra‐sensitive Simoa. Multimorbidity burden was measured by the total number of 16 chronic conditions, defined based on laboratory values, medical history, objective measures, and self‐report. We conducted linear regression to assess the associations of multimorbidity burden with plasma AD biomarkers and effect modification by race/ethnicity.

**Result:**

Median multimorbidity burden was 3 (interquartile range 2‐4), with 86% of the participants having ≥2 chronic conditions: 14% with 0‐1, 41% with 2‐3, and 45% with ≥4 chronic conditions. After adjusting for age, sex, and race/ethnicity, multimorbidity burden was significantly associated with a lower level of Aβ42/40 and higher levels of t‐tau, p‐tau181, and NfL (Table 1). The associations remained significant after further adjustment for education, physical activity, and serum creatinine, except for Aβ42/40, in which the association was attenuated. We found significant interactions by race/ethnicity (p<0.05, Figure 1), with the associations of multimorbidity with p‐tau181 and NfL being stronger among MAs and Blacks than among NHWs.

**Conclusion:**

Greater multimorbidity burden is associated with elevated p‐tau and NfL, particularly among race/ethnic minorities. This suggests that multimorbidity may be involved in tau‐related and neurodegenerative pathways of AD, as well as other peripheral clearance mechanisms. Efforts to interpret plasma biomarkers for screening purposes could benefit from considering multimorbidity and potentially tailored approaches in diverse populations.